# Corneal cell therapy: with iPSCs, it is no more a far-sight

**DOI:** 10.1186/s13287-018-1036-5

**Published:** 2018-10-25

**Authors:** Koushik Chakrabarty, Rohit Shetty, Arkasubhra Ghosh

**Affiliations:** 1 GROW Research Laboratory, Narayana Nethralaya Foundation, Bengaluru, India; 2 Cornea and Refractive Surgery, Narayana Nethralaya, Bengaluru, India

**Keywords:** Cornea, Induced pluripotent stem cells, Differentiation, Disease modeling, Cell replacement therapy

## Abstract

Human-induced pluripotent stem cells (hiPSCs) provide a personalized approach to study conditions and diseases including those of the eye that lack appropriate animal models to facilitate the development of novel therapeutics. Corneal disease is one of the most common causes of blindness. Hence, significant efforts are made to develop novel therapeutic approaches including stem cell-derived strategies to replace the diseased or damaged corneal tissues, thus restoring the vision. The use of adult limbal stem cells in the management of corneal conditions has been clinically successful. However, its limited availability and phenotypic plasticity necessitate the need for alternative stem cell sources to manage corneal conditions. Mesenchymal and embryonic stem cell-based approaches are being explored; nevertheless, their limited differentiation potential and ethical concerns have posed a significant hurdle in its clinical use. hiPSCs have emerged to fill these technical and ethical gaps to render clinical utility. In this review, we discuss and summarize protocols that have been devised so far to direct differentiation of human pluripotent stem cells (hPSCs) to different corneal cell phenotypes. With the summarization, our review intends to facilitate an understanding which would allow developing efficient and robust protocols to obtain specific corneal cell phenotype from hPSCs for corneal disease modeling and for the clinics to treat corneal diseases and injury.

## Background

Isolation of human embryonic stem cells (hESCs) from the inner cell mass of a human embryo [1] initiated the field of pluripotent stem cells and also formed the basis for developing methodologies to model human development, diseases in vitro expanding the horizons of regenerative medicine. Over time, application of hESCs for treatment modalities has been hampered due to issues pertaining to limited supply, genetic diversity of the embryos, and more importantly ethical implications over the destruction of embryos to derive hESCs [2]. These issues were alleviated to a great extent by the work of Yamanaka and colleagues on somatic cell reprogramming [3]. They demonstrated for the first time that a terminally differentiated somatic cell (human dermal fibroblast) could be re-programmed to a primordial stem cell state by introducing four pluripotency-inducing transcription factors using viral vectors. The resulting induced pluripotent stem cells (iPSCs) were similar to hESCs in their self-renewal and differentiation potential. Rapid adoption of iPSC technology demonstrated the robust nature of the reprogramming process, and iPSCs can now be generated using various gene combinations and delivery methods [4, 5]. These vast potentials of the iPSC technology have touched almost all spheres of medical biology. Ophthalmology per se has remained at the forefront of cell and gene therapy applications, for its ease in delivery techniques and outcome assays. Interestingly, a degenerative disease of the eye called age-related macular dystrophy (AMD) characterized by a progressive loss of retinal pigment epithelium (RPE) cells is the first disease candidate to gain approval for testing the clinical safety and efficacy of iPSC-derived cell technology [6]. Developments in the application of the iPSC technology in the sphere of corneal diseases have been sparse compared to retinal diseases. Two recent studies demonstrating the generation of corneal organoids [7, 8] (consisting all the cellular layers of the cornea) from hiPSCs have brought significant excitement into the field.

Corneal diseases are the most common debilitating source of visual loss that may lead to permanent blindness [9]. Although corneal-related blindness is a major health issue [10], lack of in-depth knowledge about the pathogenesis of many of the corneal diseases has hampered drug development thereby limiting treatment options. Corneal transplantation is the last resort to treat most of the corneal diseases, thereby adding a significant load on the already burdened eye banks for tissue availability. Also, corneal transplantation as a procedure has a high usage of steroids to prevent graft rejection that can lead to secondary complications [11]. Genetic studies of corneal diseases have mostly been restricted to the identification of the typical gene mutation/s [12] with little advancement towards the understanding of the cellular mechanisms involved. Moreover, most of the insights into corneal disease pathology obtained thus far are from the investigations carried out using immortalized cell lines or engineered animal models [13, 14], which are unable to fully capitulate the human conditions, thereby lacking disease relevant mechanistic insights. These critical limitations have been attributed to the lack of proper tissue context and interspecies differences, which can now be addressed by somatic cell reprogramming. The possibilities to generate corneal cells and corneal organoids from patient-specific iPSCs and also derive isogenic iPSCs lines carrying corneal disease mutations [15] (describes the generation of iPSC lines for a range of human diseases) will allow to model corneal diseases and use it as a platform to dissect the molecular mechanisms involved. Generation of corneal cells from patient-derived iPSCs will also facilitate drug discovery and the possibility to develop strategies for corneal cell replacement in a personalized manner thereby reducing the dependence on the availability of donor cornea. Combining technologies such as genome editing [16] to rectify the mutations in corneal cells generated from patient-derived iPSCs add to the potential in terms of immune-matched corneal cells for autologous transplantation.

## Potential of iPSC technology to address corneal diseases

**Fig. 1:**
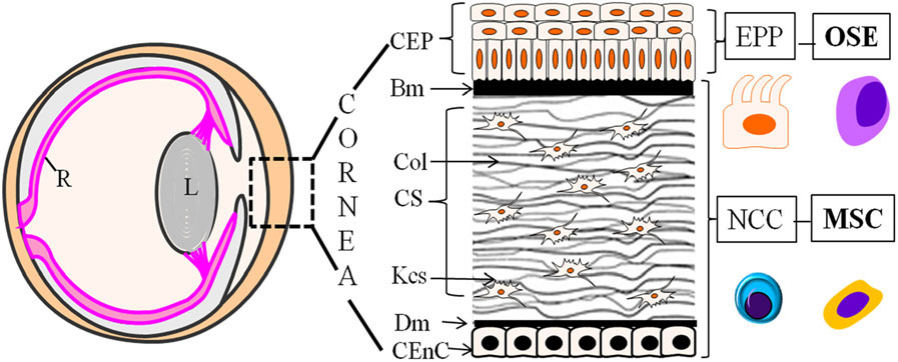
Schema of layers in the cornea and its development. The cornea constitutes of three cellular layers: the CEC, CS, and CEn and two acellular membranes. The Bm separating the CEP and CS. Dm sandwiched between CS and CEnC. The CEP is derived from the PEP originating from the OSEs. Both CS and CEnC derive from NCC which rise from the MSC

The cornea provides two thirds of the refractive power of the eye and is composed of five well-defined layers (Fig. 1), including three cellular layers separated by two acellular membranes. The phenotype of corneal diseases is seen when one or more layers of the cornea are affected. Loss of the corneal epithelial cells (CECs), the steering factor for many of the corneal diseases, is primarily due to the loss of epithelia-replenishing limbal epithelial stem cells (LESCs). Studies have shown the efficacy of LESC transplantation in limbal epithelial stem cell deficiency (LESD)-associated corneal disease [17]. However, a crucial aspect is in patients who have bilateral LESD where there is no feasibility to obtain autologous LESCs. In this scenario, transplantations are done with ex vivo-cultivated oral mucosal epithelial cells which have shown to cause detrimental vascularization and early fibrosis of the transplant in some of the cases [18]. Storage of the transplant is a key aspect which may potentially increase the clinical outcome and safety of the procedure by providing a logistical window for a phenotypic investigation [19] and planning of surgery. In their attempt to identify the effects of preservation time on proliferative potential of human limbal stem/progenitor cells, Liu et al. [20] demonstrated that long-term preservation of limbal explants caused severe disturbances of epithelial integrity along with the loss of their viability. They also reported impaired proliferation and migration of the stored LESCs when cultured in vitro. Oral keratinocytes were shown to have the potential for treating LSCD in humans and is one of the only non-limbal cell types that has been used [21]. Accumulating evidence for using oral keratinocytes as transplants for LSCD has led to efforts towards storage of these cultured cells [22]. Comparing different storage temperature, Islam et al. [23] reported the effects of storage temperature on the structure and function of cultured human oral keratinocytes. Subsequently, Utheim et al. [22] found that storage temperature also affects the gene expression pattern of the cultured human oral keratinocytes. Here, it is crucial to note that the authors observed storage temperature influencing the expression of genes involved in both the proliferation and differentiation process of oral keratinocytes extending its significance in the field. The lower survival rate of the transplanted oral epithelia in the corneal limbal regions [19] is further accentuated by the duration of storage of cultured LESCs and oral keratinocytes limiting its availability for repeat transplants which is often necessary to address some of the LESC-related corneal surface diseases. These challenges can be addressed using iPSCs which can be stored effectively upon their generation and directed to LESCs and CEC phenotype when required. Efforts towards obtaining LESCs from iPSCs have provided good results [24] thereby placing iPSCs as a promising source of transplantable LESCs. Another common affliction of the cornea is the corneal dystrophies (CD) which typically have a genetic etiology [25] and often with no options for therapy other than keratoplasty in advanced cases. Corneal diseases such as the dystrophies are a persisting global health concern with a significant economic burden since there are very limited drug-based treatments available. In addition, there are problems of graft rejection, or the transplanted tissue also being affected with the disease as the underlying cause for the pathology has not been addressed. However, for many of the CDs, the cellular signaling mechanisms involved in their pathology are still elusive. Although studies [26] have demonstrated the formation and accumulation of the mutated gene products (proteins) in most of the corneal dystrophies, little is known about the contextual molecular mechanisms involved in the formation of such deposits. Therefore, understanding the cellular context and relevant mechanisms involved in corneal dystrophy is imperative for identifying possible therapeutic interventions. Differentiation protocols continue to improve leading to robust generation of corneal cells from iPSCs, thereby providing the necessary platform to model the corneal diseases and its utilization in cell replacement therapy.

## How to generate corneal cell phenotypes from iPSCs?

**Fig. 2:**
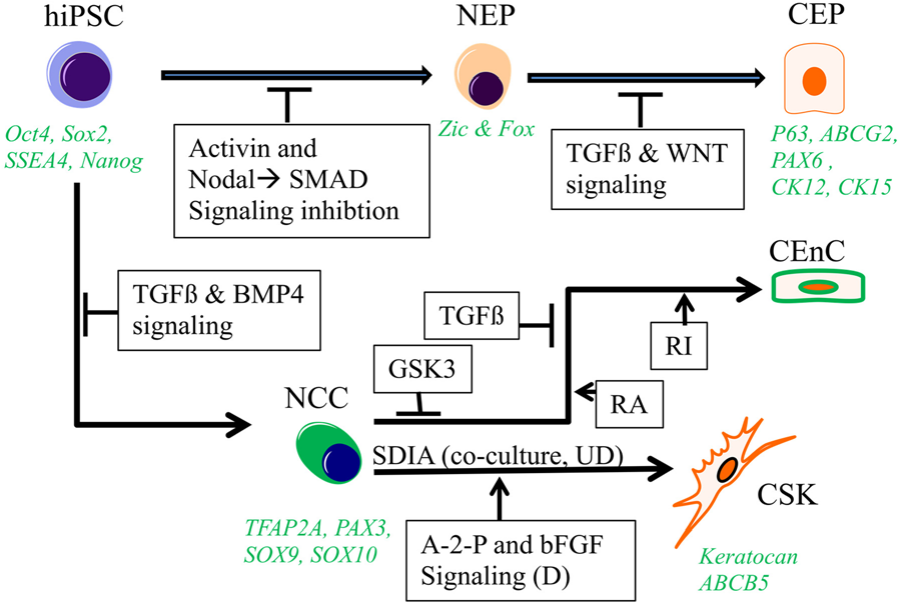
Schema of deriving corneal cell phenotype from iPSCs. Human iPSCs treated with competitors of activin, and nodal pathways result in the inhibition of SMAD signaling inducing neuroectodermal progenitor (NEP) fate by activation of Zic and Fox gene family. Subsequent directed differentiation of NEPs to corneal epithelial cells (CEPs) having expression of Pax6, ABCG2, p63, and cytokeratin 12 and 13 is done by inhibiting TGFβ and WNT signaling pathways. To obtain CSKs, iPSCs are at first directed towards NCC phenotype by inhibiting TGFβ and BMP4 signaling using SB431542 and Noggin respectively. NCCs can be differentiated to keratocan and ABCB5-positive CSKs by following a co-culture system involving PA6 stromal cells for SDIA or by following a more defined culture method utilizing the bFGF and ascorbic acid (ascorpate-2-phosphate, A-2-P) signaling pathway. ZO-1 and Na,K-ATPase-positive CEnCs (see references [68, 78] for hCEnC markers) can be differentiated from NCC following a sequential differentiation procedure where the NCCs are first treated with a GSK3b inhibitor to activate the WNT/β-catenin pathway followed by treatment with SB431542 to inhibit TGFβ-mediated SMAD signaling. RA promotes terminal CEnC differentiation inhibiting while ROCK inhibitor promotes survival and enhances functional properties of the CEnCS [83, 84]

The protocols devised for differentiating pluripotent cells to a particular cell fate has mostly relied on the developmental studies of the particular cell or organ in question. In case of the eye, our knowledge is mostly derived from the developing mouse [27] and chicken embryos [28]. These animal models have lent immense knowledge in elucidating the spatial and temporal expression of instructive molecular cues; the same knowledge in the human eye development is warranted. Thus, while we extrapolate the animal data for human application, there remains a possibility of generating non-ocular cells during the directed differentiation process. The directed differentiation approaches [29] generally involve growth factors or small molecules [30] to recapitulate the ontogeny of the cell type of interest, for example, corneal epithelium, corneal keratocytes, and corneal endothelium [31] (Fig. 2). Recent advances in 3D culture technology allow stem cells such as iPSCs to self-organize during its differentiation process resulting in an organoid, which reflects the key structural and functional properties of the organ [32]. Two recent studies demonstrated the possibility to obtain corneal organoids from hiPSCs. Foster et al. [8] in their pursuit to develop retinal organoids from human fetal fibroblast-derived iPSCs promoted an anterior neural commitment of the iPSCs using a Matrigel extracellular matrix (ECM), inhibition of Wnt signaling, and manual dissection of the developing neural vesicles followed by exposure to retinoic acid and temporally limited Notch signaling. This approach produced 3D optic vesicles and anterior neural vesicles. However, it also gave rise to translucent organoids having corneal features which upon extensive characterization were revealed to share features of the developing cornea, harboring three distinct corneal cell types with expression of key epithelial, stromal, and endothelial cell markers. In another study, Susaimanickam et al. [7] obtained corneal organoids first by differentiating the iPSCs and ESCs to the eye field primordial clusters which were manually excised for suspension culture for subsequent development of corneal organoids. The possibilities of obtaining patient-derived corneal organoids to model cornea development and corneal diseases “in a dish” hold promise for developing predictive diagnostic markers, drug testing, and personalized medicine. Although corneal organoids can serve as a powerful tool to study disease development or predict drug response, its application in corneal tissue replacement is currently limited due to its organized multi-cellular phenotype.

The pathologies of most of the corneal diseases including the dystrophies are usually limited to a specific layer of the cornea [33]. Replacement of the diseased cells with an iPSC-derived healthy corneal cell of the required phenotype (corneal epithelium, corneal keratocytes, or corneal endothelium) would be an ideal strategy to address the corneal diseases. Therefore, in this review, we discuss some such methods to derive corneal cells and tissues from hiPSCs.

## Derivation of corneal epithelial cell phenotypes from iPSCs

The integrity and homeostatic function of the corneal epithelium are crucial for maintaining the transparency and visual function of the cornea. Under homeostasis conditions, the corneal epithelium (CE) is renewed and maintained by its progenitor cells in the limbus. An injury or disease causing the loss of CE affecting corneal health and its function has therefore been a matter of interest in the ocular field. To treat the loss of CE, current therapies involve direct implantation of the limbal tissue containing LESC population from the unaffected eye when the complication is unilateral. However, limitations to such transplantation therapies arise from the risk of damaging the donor healthy eye from which the LESCs are obtained in the case of unilateral transplantations [33]. While in case of bilaterally affected subjects undergoing grafts from donors, the risk of immune rejection is often a possibility due to the allogenic nature of the transplant or due to the lack of a sufficient number of corneal epithelial cells which can repopulate the ocular surface and function optimally without being rejected [34]. Therefore, alternative therapeutic approaches are an unmet clinical need in bilateral loss of LESCs or CE. Deriving transplantable CECs or its LESCs from iPSCs [35] has tremendous potential to be the ideal option to treat CE and ocular surface diseases, but it is still a challenge as the conditions and signals to derive them in human context are inadequately understood. Most of the protocols (Table 1) for differentiating ESCs or iPSCs to CECs draw from our understanding of the ectoderm development. During embryogenesis, CE originates from the head/ocular surface ectoderm [35]. Although many of the developmental mechanisms and signaling routes remain elusive, it is known that blocking transforming growth factor (TGF)-β/Nodal and Wnt/β-catenin signaling pathways are required for head/ocular surface ectoderm development [36]. A small molecule SB-505124 and its analog the SB-431542 selectively inhibit TGF-β inducing the neural fate with the help of another small molecule—IWP-2—which functions as an inhibitor of the canonical Wnt pathway [37]. The effects of a combination of two small-molecule inhibitors, SB-505124 and IWP-2 for blocking TGF-β and Wnt/β-catenin signaling pathways together with basic fibroblast growth factor (bFGF), have been shown on the differentiation of hiPSCs towards eye precursors and further towards CECs [38]. Combining IWP-2 along with Rho-associated protein kinase (ROCK) inhibitor has been shown to drive iPSCs to the corneal epithelial progenitor (CEP)—LESC fate [39]. In another study by Ahamad et al., differentiation of hPSCs into corneal epithelial-like cells was achieved by growing the hPSCs on collagen IV matrix using primary limbal fibroblast-conditioned medium [40]. The terminally differentiated CECs expressed the CE marker cytokeratin (CK) 12 and ΔNp63α, although an exclusive marker for corneal epithelial progenitor cells is yet to be identified. The transcription factor p63, especially its isoform ΔNp63α, has been linked to the stemness and being highly expressed in the basal layers of the CE and limbus is considered as a biomarker defining successful limbal transplantation [41]. Differentiation of CECs from hPSCs has proven to be rather challenging, with most of the previously published studies relying on the use of undefined factors, such as conditioned medium [42], PA6 feeder cells, and Bowman’s or amniotic membrane [24, 40]. Protocol to derive corneal epithelial cells from iPSCs [43–46] has provided critical insights into the role of each of the exogenous factors incorporated in the culture process. For example, BMP4 has been shown to be critical in the directed differentiation process of PSCs to corneal epithelial progenitors (CEPs) [44, 47] while Hayashi et al. [24] demonstrated BMP4 treatment suppressed CE differentiation from iPSCs. Kamarudin et al. [43] recently reported differences in the activity of the endogenous bone morphogenetic protein (BMP) signaling between hiPSC lines and how it impacts their differentiation fate. They reported low endogenous level of BMP in the hiPSCs hinders their directed differentiation to CECs. Previously, Quarto et al. [48] investigating the crosstalk between BMP and TGFβ signaling revealed how their interplay affects directed differentiation of hiPSCs. Inhibiting TGFβ signaling using the small molecule SB431542, Kamarudin et al. restored the endogenous BMP pathways by changing the signaling balance in the favor of BMP signaling thereby promoting the commitment of unresponsive hiPSCs to CE progenitors. Most current studies employ a multi-step approach using defined and xeno-free culture medium along with the factors which preferentially induce CE phenotype [46]. From these studies, we learn about two crucial aspects: one being the density of cells plated for differentiation, as it affects the differentiation efficiency [44], and the second being the choice of extracellular matrix for directed differentiation. In case of differentiation of the iPSCs towards CE fate, collagen IV [47] has been demonstrated to be an ideal substrate. Interestingly, Zhang et al. demonstrated that slightly elevated CO_2_ was conductive to the differentiation of CE progenitors from hESCs [49]. However, the underlying mechanism remained unclear which points to the need for further standardization of the crucial parameters such as the compatibility of the substrate, nature of the pluripotent cells, and the media used for culturing the cells apart from the oxygen modulation. Most studies aim to use defined in vitro conditions towards generating CECs from iPSCs such that the protocols are reproducible and lead to the development of clinical grade production of corneal epithelial cells [50]. Directed differentiation of iPSCs to CECs depends on the expression of cytokeratins (CK) 12 and 13 [51] while CK3 expression evident in cell lines derived from the CE [52]. In order to improve the yields of mature CECs and to obtain a stratified cell sheet resembling the native CE, a consistent and efficient stratification method would need to be employed. It is not uncommon to detect variation in differentiation potential among different hiPSC lines [53], with donor identity and gender being among the potential sources of variation in the case of hiPSC lines [54]. Therefore, different iPSC lines from multiple sources should be rigorously tested in terms of appropriate cell morphology, gene, and protein expression.

**Table 1:** Derivation of LESCs and CECs from hiPSCs

Authors/year/reference number	Stem cell type	Cell type	Time line in days	Culture conditions	Remarks
Ahmad et al./2007/40	ESC	CEP	5	Differentiated on collagen IV (ColIV)/laminin/fibronectin-coated substrate and limbal fibroblast-conditioned medium	ColIV was found to be a better substrate compared to laminin and fibronectin.
Hayashi et al./2012/24	iPSC	CEP	100	Differentiated on gelatin-coated substrate and the latter on PA6 feeder layer (stromal-derived inducing activity) in GMEM culture medium + 10% KOSR	Human CEPs were reprogrammed to iPSC using lentivirus. iPSCs were cultured in feeder-dependent conditions.
Brzeszczynska et al./2014/42	ESC	CEP	21	Differentiated on Matrigel substrate in human limbal fibroblast-conditioned medium	Derived CEPs were characterized for P63, ABCG2, and CK expression.
Michailova et al./2014/46	iPSC	CEP	44	D1–4 suspension culture: Cnt-30 medium supplemented with TGFβ and WNT inhibitors or in basal RegES medium. D5–44 adherent cultures on collagen IV-coated substrates in CnT-30 medium supplemented with TGFβ and WNT inhibitors	Derived CEPs were characterized for P63, PAX6, CK3, CK12, and CK15.
Michailova et al./2015/38	ESC/iPSC	LESC	35	Suspension culture: cultured in ESC medium + inhibitors of TGFβ and WNT Adherent culture: cultured on ColIV-coated substrates in CnT-30 medium	Comparative proteomics reveal iPSC-derived LESCs are similar to native ocular surface epithelial cells
Hayashi et al./2016/114	iPSC	CEP	100	D0–28: on laminin substrate in differentiation medium: GMEM + KOSR + NEAA + Na-pyruvate D29–56: on laminin substrate in corneal differentiation medium	The authors mention the use of an appropriate iPSC clone is import to achieve CEP differentiation
				D57–70: CnT – PR: DMEM + keratocyte growth factor + RI D71–100: on laminin substrate in corneal epithelial maintenance medium	
Cieślar-Pobuda et al./2016/45	iPSC	CEP	21	Cultured on gelatin-coated substrate in human limbal fibroblast-conditioned medium	hDF is reprogrammed to iPSC using lentivirus. Derived characterized for P63, ABCG2, PAX6, and cytokeratin expression.
Aberdam et al./2017/47	iPSC	LESC	35	D0–4: induction medium with TGFβ inhibitor + BMP4*	Modified the culture condition to produce a propagatable pure population of iPSC-derived LEC (LiPSC). *Hayashi et al. [19] show BMP4 treatment suppressed CEP differentiation from iPSCs.
				D5–21: cultured as monolayer on collagen IV substrate in induction medium with TGFβ inhibitor + BMP4 + EGF + RI	
				D22–35: Induction medium with keratocyte growth factor + RI on collagen IV substrate.	
Garcia de la Torre et al./2017/39	iPSCs	LESCs	14	D0–1: EBs in complete essential medium 6 + inhibitors of TGFβ, WNT + bFGF D2–14: Corneal epithelial limbal-conditioned medium	Derived LESCs were validated for the expression of PAX6, P63, and cytokeratins.
Zhang et al./2017/49	ESCs	LESCs	9	D0–1: ES medium	Higher CO_2_ has beneficial effects on the differentiation of corneal epithelial progenitor cells (CEPCs) from hESCs.
				D2–9: cultured under 5%/7%/9% CO_2_	
				In keratocyte serum-free medium + DMEM/F12 on ColIV substrate	
Kamarudin et al./2017/43	ESCs/iPSCs	CEP	20	D0–1: mTesR medium + RI D2–20: compared different composition of differentiation medium with TGFβ, WNT inhibitors. On D9, cultures were plated on collagen IV-coated substrate	A two-step protocol reporting better CEP differentiation efficacy. The work [35] reveals a differential ability of hiPSC lines to generate CEPs underlined by the activity of endogenous BMP signaling pathway.
Hongsito et al./2017/44	ESCs/iPSCs	LESCs	143	D0–1: suspension culture; inhibition of TGFβ and WNT pathway; addition of bFGF and BMP-4 D2–143: monolayer culture on laminin and collagen IV-coated substrates in CNT-30 medium	Methodology to produce two clinically relevant ocular epithelial cell types from feeder- and xeno-free hPSC.

## Derivation of corneal keratocytes from iPSCs

Keratoplasty is a primary treatment option to treat many of the corneal conditions including corneal injury, corneal dystrophy, keratoconus (KC), and corneal infractions [55]. The ever-increasing number of patients needing keratoplasty has led to the shortfall of viable donor cornea [56]. The burden of viable corneas is expected to worsen in the coming years with a shift on the ratio of demand and availability. Hence, it is necessary to find alternatives such as iPSC-based therapies and strategies to generate the primary cellular components of the corneal stroma, the keratocytes. These quiescent cells are involved in the generation and maintenance of the stromal ECM, which confers transparency to the cornea [57], and their loss is often observed in KC [58]. In vivo, corneal keratocytes are limited in abundance, but under in vitro conditions, keratocytes are known to proliferate in the presence of medium supplemented with serum [59, 60]. However, exposure of keratocytes to serum in culture medium leads to fibroblast differentiation and the downregulation of keratan sulphate proteoglycan (KSPG) expression which is a unique product of corneal keratocytes [61–63]. Thus, access to these cells for modeling KC or for regenerative approaches is only possible using pluripotent stem cells such as iPSCs with the potential to differentiate into keratocytes. Currently, with no animal model for KC, efforts are being done utilizing iPSCs to model KC. Joseph et al. [64], generated iPSCs from normal and KC patients and compared their transcriptome profiles. They found significant downregulation in the mRNA expression of the genes involved in cell proliferation and cell differentiation pathways in KC iPSCs compared with the normal iPSCs. To make corneal keratocytes, the authors first drove the hiPSCs from embryoid bodies (EBs) in TeSR1 medium for 5 days after which the EBs were cultured under feeder-free conditions in keratocyte differentiation medium (KDM) constituting of DMEM/F12, FGF2, insulin, transferrin, and selenite for 7 days before obtaining keratocan (corneal keratocyte marker)-positive corneal stromal keratocytes (CSKs). FGF2 and insulin as growth factors have been previously used as components for KDM [65]. Long et al. [66] reported the inductive capability of FGF2 in KSPG production in bovine corneal cultures. Their work also demonstrated the ability of FGF2 to prevent serum-induced downregulation of KSPG which is lost with sub-culturing and is usually accompanied with the appearance of fibroblastic phenotype corroborating three independent works [56–58]. In an alternative approach, Naylor et al. [67] followed a two-step protocol to differentiate hiPSCs to corneal keratocytes. In the first step, they differentiated the iPSCs to an intermediate neural crest cells (NCCs) stage, which were then differentiated to corneal keratocytes. For NCC production, the authors tested and compared two established NCC protocols [68, 69] and found the protocol from Chambers et al. [62] more efficient for their purpose (Box 1). The iPSCs were cultured on Geltrex-coated plates in the modified TESR1 medium in the presence of ROCK inhibitor Y-27632 for the first 24 h with the derivation of NCCs in about 6 to 8 days of culture. The authors employed two separate approaches for subsequent generation of CSKs from the NCCs. In the first approach, the NCCs were cultured as a substratum-independent pellet in KDM containing FGF2 and ascorbic acid 2-phosphate for 21 days to obtain CSKs. In their other approach, the NCC was cultured on cadaveric corneal-scleral limbal rims as natural scaffold also providing the necessary cues to direct differentiation to the CSK phenotype. They found their second approach involving the sclera rims more efficient in generating CSKs which shared the typical phenotypic characteristics of their in vivo counterpart.

These studies highlight the necessity for stepwise paradigms, where the iPSCs are first driven to the intermediate neural crest (NC) stage followed by a robust directed differentiation to CSKs. An interesting attribute of culturing CSKs which has been applied in devising iPSC-based protocols to derive CSK is their ability to form aggregates and maintain their phenotype when deprived of substratum attachment [67]. Funderburgh et al. [70] demonstrated keratocytes are aggregating into spheroids resulting in a stable and viable population of mature keratocytes with the ability to secrete ECM proteins [71]. Additional studies [72–74] from the same group elegantly elucidated the two-step protocol towards the differentiation of hESCs to CSKs. At first, NC fate was induced by culturing the ESCs on PA6 feeder layer for stromal-derived inducing activity (SDIA). The NCCs were reported to be generated by 6 days of culture and validated by their expression of the neural crest genes such as NGFR, NTRK3, and MXS1. Subsequently, positively selected (based on the expression of cell surface markers CD271 and p75NTR) NC precursors were further differentiated to CSKs in KDM. These NC-derived CSKs were shown to demonstrate one of the key functions of corneal keratocytes, i.e., to secrete high molecular weight proteoglycan such as keratan sulphate and keratocan. The CSKs generated from hPSCs from both hESC and hiPSCs (irrespective of their somatic origin) [66, 68] have been shown to express mature corneal keratocyte markers as their in vivo counterpart. Most of the protocols detailing the generation of CSK from hPSCs (Table 2) adhere to a certain time line for the process of the directed differentiation. However, little is known regarding the phenotypic stability of the hPSC-derived CSKs. The KDM used in the studies involving the generation of the CSKs from hPSCs (hESCs and hiPSCs) is serum-free which is critical for the retention of the corneal keratocyte phenotype. On the other hand, the presence of serum has been reported to convert the keratocytes to fibroblast phenotype and enhances its viability at the cost of ECM production [60, 65]. The possibility to differentiate hiPSCs to bona fide human CSKs has significant implications for modeling corneal diseases and for cell replacement therapy, where CSKs have shown robust potentials in animal studies [75]. However, the characteristic cellular plasticity of CSKs in culture is to be taken into consideration while developing strategies involving these cells in human cell therapy.

**Table 2:** Derivation of CSKs from human pluripotent stem cells

Authors/year/reference number	Stem cell type	Derived cell type	Time line in days	Culture conditions	Markers evaluated	Remarks
Joseph et al./2016/64	iPSCs	CSK	20	D0–5: EBs in Tesr1 media D6–14: KDM	Keratocan	Model corneal disease using patient-derived iPSCs
Naylor et al./2016/67	iPSCs	CSK	30	D0-D8: cultured on Geltrex substrate in ES medium + RI	ALDH1A1, ALDH3A1, keratocan, and CHST6	hiPSCs to keratocyte cells
				D9–30: KDM		
Chan et al./2013/73	ESCs	CSK	12	D0–6: on PA6 feeders in ES medium	Keratocan; Aldh3a1	At D6, keratocyte precursor cells selected by NGFR expression
				D7–14: KDM		
Hertsenberg et al./2015/74	ESCs	CSK	23	D0-D7: induction on PA6 feeder layer (for SDIA) D7–21: KDM	Keratocan; keratan sulphate	Differentiated first to NCSC then sorted by NGFR and cultured in KDM

## Derivation of corneal endothelial cell from iPSCs

Human corneal endothelium (hCEn) which originates from cranial NC cells is approximately 4 μM in thickness. This monolayer of hexagonal hCEn cells lining the Descemet’s membrane of the posterior cornea maintains the dynamic fluid and nutrient balance across the stroma [76]. Being highly metabolic these cells are sensitive to changes in nutrients, altered internal protein function and reactive to various stresses making them susceptible to degeneration [77]. The loss of corneal endothelial cells (CEnCs) is detrimental to the corneal function and is the reason for many of the corneal pathologies such as Fuchs and congenital hereditary endothelial dystrophy (CHED). Furthermore, the hCEnCs have very limited proliferative ability in vivo, and their density gradually decreases with age from approximately 4000/mm^2^ post-natal to 2000/mm^2^ in older adults [78, 79]. As the damage or loss of hCEnCs is irreversible, treatment is restricted to transplanting the full thickness cornea (penetrating keratoplasty) or the endothelial cell layer alone from cadaveric donors. Culturing hCEnCs ex vivo is technically challenging as the basis for its appropriate molecular basis of maintaining functional identity is not well established. Currently, efforts are being made to properly characterize in vitro hCEnC culture to overcome poor donor availability and as a step towards cell replacement therapy [80]. Additionally, due to the potential for immune rejection, novel strategies are required to meet this unmet clinical challenge. Patient-derived iPSCs and the possibility of generating CEnCs present many advantages that can address the aforementioned limitations from availability to immune rejection. However, development of protocols for the directed differentiation of iPSCs to CEnCs in vitro is still at an early stage due to the limited insight into the hCEn development process [81]. The hPSCs were at first driven to embryoid body (EB) formation emulating NC fate using all-trans retinoic acid (RA) treatment. This was followed by a second induction using CEnC- or lens epithelial cell (hLE)-conditioned medium (CM) to ultimately generate CEnC-like cells (Chen et al. 2015). Song et al. [82] introduced a modified two-stage differentiation method to convert hPSCs to NCCs first and then direct differentiation to CEnC-like cells. The CEnC-like cells were treated with bovine CEnC conditional medium to condition the development and maturity of the hESC-derived CEnC cells. The study compared the transcriptome of hESC-derived CEnC-like cells with human primary fetal and adult CEnCs. This comparative investigation clearly demonstrates that the cells although having different origin express TRIT1, HSPB11, and CRY1 which can be used as molecular markers to identify stem cell-derived hCEnCs. Using defined medium condition, Hatou et al. [83] reported the induction of functional tissue-engineered corneal endothelium (TECE) from mouse and human cornea-derived progenitor cells (COPs) derived from the adult corneal stroma. Medium containing TGFβ2, glycogen synthase kinase (GSK) inhibitor, and RA was used to derive the TECE. The group in their recent study [84] demonstrated skin-derived precursors (SOPs) as a source of corneal endothelial progenitors since access to hCOPs is limited due to their small size in the cornea and limited proliferative capability. Furthermore, using autologous COPs is also unreasonable due to irreversible damage to the donor’s eye. In both their studies, the authors show the efficacy of the GSK3 inhibition and activity of TGFβ towards inducing CEnC fate. Also, it should be noted that the source of the primary cells (COPs and SOPs) is of NC origin as is the CEnCs, and the study elucidates the role of small molecules in signaling towards specific cellular fate. Zhang et al. [85] reported derivation of CEnC-like cells from hESCs through the periocular mesenchymal precursor (POMP) phase. Here, terminally differentiated CEnC-like cells were obtained by means of a transwell co-culture system with hESCs and human corneal stromal cells. The generated CEnC was then characterized extensively concluding that the CEC-like cells derived from hESCs displayed characteristics of native human CEnCs. A similar approach was followed to construct a full-thickness artificial cornea substitute in vitro by co-culturing LEC-like cells and hCEn-like cells derived from hESCs on acellular porcine cornea matrix (APCM) scaffold [86]. McCabe et al. [87] followed a two-step protocol for generating hCEnCs from hESCs, drawing from the histogenic origin of hCEnCs from NC. They utilized a feeder-independent protocol involving inhibition of the SMAD pathway (using dual SMAD inhibitors SB431542 and NoGGIN) for rapid generation of hCEnC under controlled conditions thereby making it more relevant for clinical applications. Recently, Zhou et al. [31] have delineated a multi-step differentiation protocol of iPSCs and hESCs to hCEnCs (Table 3). They first primed the iPSCs and hPSCs (hESCs-WA9) for a couple of days in a priming medium containing N2 and B27 supplements along with bFGF and non-essential amino acids (NEAA). Subsequently, the primed cultures were modulated by inhibiting SMAD, BMP, and Wnt pathway to generate eye field stem cells (EFSCs). The EFSCs were further differentiated hierarchically to a NC phenotype by inhibiting GSK3 signaling and in the presence of N2 and B27 supplements along with ascorbic acid. The NCs were plated at low density on the fibronectin-coated substrate and cultured in medium containing SB431542 and the ROCK inhibitor. This protocol for hCEnCs therefore takes into account the possible interplay of molecular signals in eye development. Another aspect which needs to be investigated is the potential scaffold or carriers of the endothelium monolayer for transplantation since transplantation of hCEnCs is the only way to manage advance CEnCs dysfunction [88, 89]. Such transplant requirements with ever-increasing demand is a significant threat to the tissue supply, and a donor tissue crisis is imminent. Lack of insufficient number of cells and heterogeneity in culture conditions, transplantation method, and issues of rejection adds to the viability of the overall procedure to address hCEnCs dysfunction [90, 91]. iPSC-derived hCEnCs address most of these concerns. Implantation of the stem cell-derived hCEnCs delivered without transferring cells on a membrane cell carrier is being devised to enhance the efficacy of the implant (see review [92]). The biological property of ROCK inhibitor showing excellent efficacy in hCEnC regeneration in vivo [93] and expansion of cultured hCEnCs [88, 94] (by manipulating the cell adhesion properties [93, 95]) can be harnessed in the latter steps of the multi-step hCEnC differentiation protocol. The major advantage of deriving CEnCs from iPSCs will be to reduce and possibly eliminate corneal donor tissue shortages because the transplanted cells can be grown in a laboratory and used to treat several patients instead of only one patient.

**Table 3:** Derivation of CEnCs from hPSCs

Authors/year/reference number	Stem cell type	Derived cell type	Time line in Days	Culture conditions	Remarks
Zhang et al./2014/86	ESCs	CEnCs	25	D0–9: EBs was placed on ECM-coated substrate in basal medium D10–25: transwell culture in CEnCM	Report the derivation of CEnC-like cells hESCs through the POMP
McCabe et al./2015/88	ESCs	CEnCs	10	D0–3: ESCs cultured in ES medium + SMAD inhibitors (Noggin, SB43152). D4-D10: ES medium + PDGF + DKK2	Global gene analysis revealed the ES-derived CEnCs similar to their in vivo counterparts
Zhao et al./2016/31	ESCs/iPSCs	CEnCs	D27	D0–2: cultured in priming medium D3–9: EFSC generation D10–18: NCSC derivation D19–27: NCSCs differentiated to CEC	Generate CEnC from PSCs under defined culture conditions following a multi-step differentiation process
Song et al./2016/83	ESCs	CEnCs	30	D0–14: NCC induction D15–30: CEnC derivation	Compared transcriptome of ESC-derived CEnCs to in vivo counterpart
Zhang et al./2017/87	ESCs	CEN	25	D0–25: culture conditions as mentioned by Zhang et al. 2014	Developed strategy for the construction of TECS by co-culturing ESC-derived LEC and CEnCs

## Translational challenges of using iPSC-derived corneal cells

Though iPSC technology has huge potential for regenerative medicine and disease modeling, it faces many challenges and limitations which require further in-depth understanding of the cellular reprogramming differentiation processes (Table 4). One of the major caveats of the iPSC technology is the low efficiency of iPSC generation and the variability in maintenance and differentiation of a mature cell of interest [96, 97]. A significant advancement is being made towards more efficient methods to derive iPSCs, which includes media formulations, substrates, and small molecules all of which promote better reprogramming efficacy and iPSC turnover [5, 98, 99]. The field has evolved from using integration-dependent viral system to reprogram integration-independent systems [100, 101]. In spite of the inherent drawback of the integration-dependent system towards somatic cell reprogramming, the higher reprogramming efficiency of the method leads to its appeal [102] and utility [103]. Another issue of the current cellular reprogramming technology is the huge variability in the iPSC characteristics such as its self-renewal capacity, expression of pluripotent genes, retention of epigenetic signature of the parental somatic cell, the differentiation potential, and genomic stability (see reviews [104, 105]). The magnitude of variations manifests as a potential challenge to using iPSC and iPSC-derived cells to model human phenotype and disease. Somatic heterogeneity can occur in iPSC lines [106, 107] during the reprogramming, and subsequent differentiation process [108] can interfere in the development of the cellular phenotype and functionality. Studies investigating these aspects [109–111] have shed significant light on the relationship between the genetic background of individuals and its association with the molecular expression phenotypes of the reprogrammed cells.

**Table 4:** Translational challenges of using iPSC-derived corneal cells in disease modeling and therapy

Process	Challenge	Solutions
Somatic cell reprogramming	Genomic stability	Using non-integrating (sendai virus, episomal vectors, small molecules) methods for reprogramming, karyotyping before reprogramming, optimizing culture conditions
	Low efficiency	Epigenetic modifiers, e.g., HDAC inhibitors, and stimulatory factors, e.g.., p53i, miRNA, signaling agonist and antagonists [134]
iPSC-derived corneal cells	Improper differentiation/genomic stability	Developing appropriate protocols (Tables 1, 2, and 3) and optimizing culture conditions, robust screening, and characterization criteria
	Genetic variability (inter- and intra-clonal)	Genome editing/isogenic lines/big sample size

The iPSCs and corneal cells differentiated from them have a significant risk of genomic instability due to the extended in vitro culture periods required [46, 112, 113]. Genomic instability of the differentiated cell phenotypes generated from iPSCs is a challenge for disease modeling and even more so for their clinical applications in cell replacement therapy. One way to address the unavoidable mutations in such long-term iPSCs and differentiated corneal cells is to validate and bank early passages of the iPSCs [114, 115]. Additionally, stringent quality control requirements can be incorporated at every step of the characterization process during differentiation [116].

Use of iPSC-derived corneal cells in the clinical application has multiple challenges, which includes derivation of clinical grade cells, potential tumorigenicity of transplanted cells, and immune-acceptance of transplanted cells. However, there are inherent advantages and disadvantages considering autologous or allogenic iPSC-derived corneal cells for cell replacement therapy. By passing the issue of immune rejection is the primary advantage of the autologous iPSC-derived corneal cells. However, the crucial challenge that would need to be addressed for the utility of such truly autologous iPSC-derived corneal cells is the time required to generate such individual iPSC line. The iPSC generation time which ranges in terms of weeks to months depends on the multitude of factors such as the age of the donor, phenotype of the somatic cell to be re-programmed, reprogramming method, and culture conditions. Additional expenditure of time and cost will be needed for the selection and characterization of the individual iPSC clones, and their derivatives significantly increasing the cost of therapy. A special advantage with most of the corneal diseases being chronic in nature is the availability of sufficient time to strategize and perform the necessary processes for generating corneal cells from autologous iPSCs. However, it is important to note that autologous cells carrying gene defects will need to be corrected necessitating more time and cost for the process and characterization of the derived cells and to be taken into consideration while devising strategies for the generation of corneal cell phenotypes from iPSCs. For accessibility and application of iPSC-based cell therapy, it is important to address the challenge for keeping the costs affordable yet have a robust derivation process of corneal cells within an acceptable time line. The Japanese study [6] using autologous iPSC-derived RPE cells to treat AMD suggests robust Good Manufacturing Practice (GMP)-compliant protocols for culturing of iPSCs and their derivation to RPE. The pluripotent nature of the iPSCs also raises the concern that any undifferentiated pluripotent stem cells remain in the final clinical product could increase the risk of tumor or teratoma formation after transplantation. This possibility is further underscored by the recent observation of potential tumorigenic mutations in some of the clinical-grade iPSC lines derived from one AMD patient as part of a clinical study at the RIKEN Institute in Japan [117]. The other emerging aspect is the immune response directed at autologous iPSC-derived cells which have been well reviewed by Scheiner et al. [118]. A recent clinical study involving ESC-derived RPE cells addressed the safety concerns of the ESC-derived cells and provides evidence in favor of ESC-derived cell therapy to treat AMD [119]. Compared to the allogenic source of pluripotent stem cells, the autologous derived corneal cells will require a considerable amount of time and cost to generate them in an individual manner which can be circumvented by generating iPSC lines from the selected distribution of allelic frequencies of HLA phenotypes in the given population. This approach will address the overall benefit to cost restrictions of autologous derived corneal cells [120]. Currently, it is surmised that a relative number (in the hundreds) of such HLA-matched iPSC lines would be sufficient [121, 122] for setting up an iPSC bank which can be a source for deriving corneal cells. However, it should be noted that banking HLA-matched iPSC lines would require a significant investment of efforts, time, and money compared to an allogenic approach which involves a couple of hPSC lines for generating the corneal cells. Furthermore, additional characterization in addition to HLA typing such as mutational profiling of the iPSCs will help to select the appropriate iPSC line for deriving the corneal cells for therapy.

Another aspect which favors the proponents of allogenic PSC source for generating corneal cells is the eye being considered an immune-privileged site due to its relative self-containment due to the barriers that keep cells from migrating both from inside or outside to other parts of the body. With progress in the field providing us with deeper insights into the mechanisms of cellular reprogramming and their induction to specific corneal cell lineages and the stability of the phenotypes will allow surmounting the concerns and paving possibilities for their utilization in the clinics.

## Challenges in modeling corneal diseases using iPSC derived corneal cells

Though iPSC technology has huge potential for disease modeling, it faces many challenges [123] which may hinder its ability to model some diseases. The conversion of somatic cells to iPSC by cellular reprogramming does involve rejuvenating the somatic cells, and conferring pluripotency capabilities [124] where epigenetic remodeling achieved by DNA methylation and histone modifications play a critical role in the global transcriptional regulation during reprogramming [125]. The epigenetic variations due to residual somatic memory [126] exist among human iPSC lines and play a critical role deciding their fates during their directed differentiation and their capacity to differentiate to specific lineages [54].

Here, it is important to note that the epigenetic changes between the somatic cells and the derived iPSCs may obscure the retention of the disease phenotype for disorders that involves epigenetic modification such as imprinting disorders or sex-linked disorders or for diseases with mixed etiologies. One potential limitation is the genetic variability between different patients or clones derived from the same patient [127] which can affect many of the critical factors such as its differentiation potential. One strategy to reduce variations within the disease phenotype is to increase the overall sample size. Reduction in the inter-clonal variability within the iPSCs to derived corneal cells can be achieved by following the strategies such as differentiating corneal cells from multiple iPSC clones having the same genetic background. Inter-individual variability can be addressed by comparing iPSCs generated from multiple clones per donor across different patients and control individuals. In recent years, the field of gene editing has progressed rapidly with the advancement of the clustered regularly interspaced short palindromic repeats (CRISPR)/Cas technology allowing easy manipulation and gene editing of iPSCs [128]. Using the gene editing tools, isogenic control and disease iPSCs can be generated by introducing the mutation/s implicated in the specific corneal diseases. Additionally, matched iPSC lines can be generated from affected and unaffected individuals from the same family. In such cases, an isogenic iPSC line can be further created from the affected patient line by utilizing gene editing to correct the mutation(s). Gene editing of isogenic iPSC clones [129] will address the inter-individual variations in the genetic backgrounds in the patient populations reducing potential individual specific and epigenetic influences on the disease phenotype. Such models can therefore make personalized and tailored treatment for the individual a close possibility. The genome editing approach should mitigate any variability due to the differences in patient genetic background since the genetically engineered cells would have been derived from the same source, provided the editing process remains specific and does not introduce non-specific genomic changes. Therefore, the application of genome edition in iPSC-based disease modeling will allow obtaining insights into the pathological pathways involved in corneal dystrophies thereby enabling identification of therapeutic targets to address the disease pathology.

## Conclusions and future perspectives

Derivation of iPSCs and differentiation to corneal cell types in a personalized manner would be an asset to venture upon in order to achieve customization of patient-specific therapy. Though the cost of multiple quality control parameters at key points of the iPSC derivation, clone characterization, and differentiation process remain expensive, the establishment of a robust technique along with the development of commercialization of “kit”-based tools can make the process affordable. Personalized iPSC-derived therapy for corneal tissue replacement is underscored by the presence of pathological genetic mutations that can be addressed by constructing population-based mutation databases with strong clinical phenotype correlation. This would eliminate the mutation screening step to some extent thereby moving forward with gene editing step. The limitation of personalized iPSC-based cell therapy for corneal diseases can be circumvented by a slightly different and perhaps logistically and financially less burdensome strategy of developing universal donor iPSC lines which can be immune-match up for a higher percentage of the population [121]. In present, scenario protocols seem to be using diverse components activating/blocking multiple signaling pathways limiting the reproducibility. It can very well be envisaged that in order to have a wider clinical applicability, it is necessary to have a standardized robust protocol with a xeno-free minimalistic approach that can be practiced with ease. Transplantation of iPSCs has been shown to alleviate cerebral inflammation and neural damage in hemorrhagic stroke [130]. The key challenge that would need to be addressed towards the clinical application of iPSC-derived corneal cells is enhancing their survival in the inhospitable environment due to the underlying disease. A robust regime such as blocking the death signaling pathways of the cells using pro-survival cocktails, pre-conditioning the iPSC-derived cells prior to transplant, and using bioengineered scaffolds or matrices which can enhance cell survival and functions would be necessary to optimize survival of the transplant. Harnessing the potential of iPSC-derived corneal cells for clinical application will require surmounting the challenges of graft survival. This challenge can be addressed by preclinical studies involving knockouts and transgenic animals and with the development of technologies to monitor the transplant. To prevent rejections of human cells in the animal models, immune-suppressed or immune-compromised animals should be considered. In this direction, humanized animal models, mice in particular, have provided significant insights in immunology [131], and efforts are being given to generate a humanized model of corneal diseases [132] which can be used to evaluate the efficacy of iPSC-derived corneal cells destined for clinical application.

To conclude, despite these promising results, more research is needed for understanding and addressing the risks involved in using cells de-differentiated from iPSCs which include right from the process of iPSC generation to its differentiation and its later utilization. A tri-party amalgamation involving the researcher, clinician, and an industry partner would achieve for providing affordable and reproducible results in patients with corneal diseases. Here, we provide a review of the application of the iPSC technology to generate corneal cell phenotypes for modeling corneal diseases and allow interrogating the genotype-phenotype relationship in a tissue-context manner. These insights would lead to the identification of possible newer molecular targets in the disease-causing pathway which can be modulated for therapy. Furthermore, in the near future, in vivo-corrected corneal cells from patient-derived iPSCs can find applications for cellular transplantation to address corneal diseases.

## Box 1

Chambers et al. [62] and Lee et al. [63] previously demonstrated the requirement for the initial induction of an intermediate NCC stage of hPSCs (hESCs and hiPSCs) prior to deriving cells of mesenchymal origin. Both these groups derived NCC from both hECSc and hiPSCs by following two independent protocols harnessing a feeder-free system along with the incorporation of small molecules such as SB-431542, a TGF-β inhibitor [133], LDN-193189, and CHIR99021 (BMP pathway modulators) and Noggin (Wnt pathway inhibitor). Interestingly, Lee et al. reported incorporating the ROCK inhibitor Y-27632 in MTESR1 medium for differentiating hPSCs to NCCs in about 28 days of the culture period. While it took about 11 days to differentiate both hESCs and hiPSCs to NCCs according to Chamber’s et al. Difference between the hESCs and hiPSCs with respect to their NCC differentiation capability was not reported in both studies indicating the similarity of the pluripotent cells of either origins. However, these two studies highlight the role of small molecules and signaling cues provided in the hPSC to NCC differentiation window.

## Abbreviations

AMD: Age-related macular dystrophy
APCM: Acellular porcine cornea matrix
bFGF: Basic fibroblast growth factor
Bm: Bowman’s membrane
BMP: Bone morphogenetic protein
CE: Corneal epithelium
CEC: Corneal epithelial cells
CEnCs: Corneal endothelial cells
CEPs: Corneal epithelial progenitor
CHED: Congenital hereditary endothelial dystrophy
CK: Cytokeratin
CM: Conditioned media
COPs: Human cornea-derived progenitor cells
CRISPR: Clustered regularly interspaced short palindromic repeats
CRY1: Cryptochrome circadian regulator 1
CS: Corneal stroma
CSK: Corneal stromal keratocytes
Dm: Descement’s membrane
EBs: Embryoid bodies
ECM: Extracellular matrix
EFSCs: Eye field stem cells
EPP: Epithelial precursors
GMP: Good manufacturing practice
GSK: Glycogen synthase kinase
hCEnCs: Human corneal endothelial cells
hESCs: Human embryonic stem cells
hiPSCs: Human-induced pluripotent stem cells
hLE: Human lens epithelial cells
hPSCs: Human pluripotent stem cells
HsPB11: Heat shock protein family B (small) member 11
KC: Keratoconus
KDM: Keratocyte differentiation medium
KSPG: Keratan sulphate proteoglycan
LESCs: Limbal epithelium stem cells
LESD: Limbal epithelium stem cell deficiency
MSC: Mesenchymal stem cells
NC: Neural crest
NCCs: Neural crest cells
NEAA: Non-essential amino acids
NGFR: Nerve growth factor receptor
OSE: Ocular surface ectoderm cells
Pi3k: Phosphoinositol 3 kinase
POMP: Periocular mesenchymal precursor
RA: Retinoic acid
ROCK: Rho-associated protein kinase
RPE: Retinal pigment epithelium
SDIA: Stromal-derived inducing activity
SOPs: Skin-derived precursor
TECE: Tissue-engineered corneal endothelium
TGF: Transforming growth factor
TRIT1: tRNA isopentenyltransferase 1
